# Greening the curriculum: challenges in bringing environmental sustainability to pharmaceutical education

**DOI:** 10.3389/fmed.2026.1770602

**Published:** 2026-02-10

**Authors:** Cristina M. M. Almeida

**Affiliations:** iMed.UL (Institute for Medicines and Pharmaceutical Sciences), Faculty of Pharmacy, University of Lisboa, Lisboa, Portugal

**Keywords:** drugs, environmental, green pharmacy, healthcare system, pharmaceutical education, pharmacists, sustainability

## Abstract

Environmental sustainability assumes a crucial dimension in modern pharmaceutical care, as the production, distribution, dispensing, and disposal of pharmaceutical products significantly contribute to environmental pollution and pose long-term health risks to the public. Pharmacists, as medication experts, play a central role in mitigating these risks through safe medication management, patient counseling, and advocacy for sustainable practices, working in close collaboration with prescribers and other healthcare professionals. It's essential to incorporate environmental sustainability into pharmaceutical curricula to prepare pharmacists for these challenges. Improper manufacturing and disposal of pharmaceuticals can contaminate soil and water, leading to antimicrobial resistance (AMR) and ecological disruption. Real-world examples, such as the detection of active pharmaceutical ingredients (APIs) in natural waters, biota, and drinking water, underscore the need to address these challenges. By embedding sustainability in education, future pharmacists develop competencies in green chemistry principles, medication review and optimization, formulary stewardship, and guidance on safe drug disposal, thereby strengthening the sustainability of the healthcare system. Several initiatives worldwide emphasize the practical necessity of this academic training. This dynamic relationship ensures that pharmacists act as both guardians of patient health and stewards of environmental wellbeing.

## 1 Introduction

The growing complexity of 21st-century environmental, social, and health challenges requires that contemporary society urgently transform its development models. Climate change, biodiversity loss, water pollution, and ineffective waste management pose significant threats not only to the environment but also to human health and global equity. Against this backdrop, the concept of sustainability is emerging as a key driver for public policies, economic strategies, and cross-disciplinary scientific approaches. Rather than being on the sidelines of this transition, the health sector occupies a central position: it is affected by the consequences of environmental crises, and it contributes to their intensification, both directly and indirectly (1).

However, concerns regarding the environmental determinants of health have deep historical roots. As early as the 1960s, Rachel Carson's critique of the widespread use of dichloro-diphenyl-trichloroethane highlighted the interconnected impacts of pharmacological interventions on ecosystems, animal life, and human health, core principles later formalized within One Health and planetary health frameworks (2). Similarly, early Science–Technology–Society educational approaches, such as those developed by Brinckerhoff, emphasized ecological awareness and societal responsibility, anticipating contemporary calls to integrate environmental sustainability into health and medical sciences (3). Among all the areas of the healthcare system, the pharmaceutical sector is particularly relevant for environmental sustainability. The industrial production of medicines, their increased consumption, and the subsequent improper disposal of pharmaceutical substances have had a significant impact on ecosystems. Pharmaceutical waste has been detected in both surface water and groundwater, impacting aquatic organisms and compromising water quality and safety. Meanwhile, pressure on the natural resources used in pharmaceutical production raises critical questions about the sector's efficiency, circularity, and environmental responsibility (4). These impacts are not merely downstream consequences of use and disposal, but also the result of anticipatory choices made throughout the pharmaceutical lifecycle, from molecule design and manufacturing processes to prescribing practices and formulary inclusion. Therefore, it is crucial to reconsider the pharmacist's role. While traditionally associated with ensuring the efficacy, safety, and quality of medicines, pharmacists today and in the future are required to expand their remit by integrating environmental concerns into their professional practice. The transition to a more sustainable pharmaceutical activity requires technological and logistical changes, but above all, a shift in mindset, attitude, and skill set. At this juncture, academic training plays a pivotal role (5). Pharmacists increasingly contribute to anticipatory decision-making processes, including participation in drug development discussions, regulatory assessments, and the critical evaluation of therapeutic alternatives with differing environmental footprints (6).

As part of the training of future professionals, higher education in Pharmaceutical Sciences must incorporate robust, critical, and integrated environmental awareness. This awareness goes beyond merely transmitting technical content on waste or environmental toxicity; it also involves promoting environmentally responsible professional ethics. This prepares students to analyze the ecological impacts of their decisions, educate users, collaborate with public policies, and actively participate in mitigating the environmental risks associated with medicines. Ecological literacy is therefore a vital skill for contemporary pharmacists, in line with recommendations from the World Health Organization and the International Pharmaceutical Federation for training “climate-smart” professionals to promote resilient health systems (7–10). Such literacy is essential to enable pharmacists to meaningfully contribute to early-stage sustainability assessments, guiding choices about which pharmaceutical substances should be prioritized, modified, or, where appropriate, phased out.

Systemic thinking is also necessary to address the complexity of health, disease, sustainability, and pharmaceutical activity. Consequently, it is needed to integrate into the pharmacists' curriculum, in addition to the core courses associated with the biological and chemical determinants of health, other determinants, namely, environmental, social, economic, and cultural (from a systemic perspective), and to develop communication tools that are often neglected. In this approach, there must also be greater communication about the values and ethics underlying pharmaceutical activity and health systems (10, 11). This systemic perspective is particularly relevant for reassessing the traditional benefit–risk paradigm, which has historically focused on patient safety and therapeutic efficacy while largely neglecting environmental risk. Incorporating environmental risk into this balance, when evidence is available and relevant, represents a forward-looking, precautionary approach that aligns pharmaceutical practice with broader public and planetary health goals (12).

Sustainability training requires an innovative, interdisciplinary curriculum that aligns with the principles of planetary health. The pedagogical approach should favor systemic thinking, critical analysis, ethical decision-making, and interprofessional collaboration—all of which are increasingly valued skills in complex and interdependent health contexts. Environmental awareness is therefore not an additional topic, but a core component of professional competence. Without the ability to anticipate long-term environmental consequences and evaluate trade-offs across therapeutic options, pharmacists will be ill-prepared to participate in strategic decisions shaping the future of medicines.

The sustainable approach is intrinsically linked to the 17 Sustainable Development Goals (SDGs) established by the United Nations, serving as a vital driver of the 2030 Sustainable Development Agenda (13). SDG 3, “ensure healthy lives and promote wellbeing for all at all ages,” and SDG 4, “quality education,” are directly linked to pharmaceutical education. The pharmaceutical sciences are inherently part of SDG 3, which is directly and indirectly connected to the other SDGs. Even at first glance, the impact of pharmaceutical sciences and the associated challenges must be considered as equally interrelated. As a transformative approach to learning, sustainability education empowers individuals with the knowledge, skills, values, and attitudes necessary to contribute to a more sustainable and equitable world. Within this framework, SDG 4 is critical, as it emphasizes the need for inclusive and equitable quality education that fosters lifelong learning opportunities for all. By promoting competencies in critical thinking, problem-solving, and active participation in sustainability initiatives, sustainability education becomes an essential tool for achieving not only SDG 4 but also the broader set of interconnected goals that define global sustainable development efforts (10, 11).

In this context, integrating real-life sustainability experiences into academic programs is crucial for preparing future professionals to address the complex global challenges they will face. Sharing practical examples and interdisciplinary approaches within the educational community enhances students' understanding of how sustainability principles apply in real-world contexts. Importantly, such experiences also expose students to real decision-making dilemmas, such as evaluating therapeutic necessity, comparing alternatives with different environmental profiles, and understanding how formulary decisions can influence both population health and environmental outcomes.

This study exemplifies such an effort by presenting the design and outcomes of a course that embeds corporate sustainability (CS) in undergraduate education. Delivered collaboratively by a consortium of professors to students from various universities and regions, the course highlights key features and learning outcomes that illustrate how higher education can effectively foster sustainability-oriented mindsets and professional competencies.

Therefore, integrating sustainability into pharmaceutical sciences education requires more than just adapting the curriculum; it necessitates a structural response to the demands of a changing world.

The sustainable development of the pharmaceutical sector relies on training professionals who can combine technical knowledge with environmental awareness, ethical responsibility, and anticipatory decision-making. Recognizing and strengthening the role of pharmacists in evaluating environmental risk alongside therapeutic benefit is a strategic priority for health education and for the responsible governance of medicines throughout their life cycle.

For a comprehensive approach to this topic, this review was divided into six sections after this brief introduction (1), to an overview of current issues relative to pharmaceutical education and sustainability; (2) fundamentals of environmental sustainability; (3) the role of pharmacists in environmental sustainability; (4) pharmaceutical curriculum and sustainability; (5) best practices for sustainable action; (6) challenges and perspectives for the future; and (7) conclusions. Therefore, the objective of this review was to analyze existing literature and theoretical models linking sustainability and pharmaceutical education and to explore the potential impact of sustainability-oriented curricular reforms on future pharmaceutical practice and public health outcomes.

## 2 Fundamentals of environmental sustainability

Environmental sustainability is defined as the ability to ensure the balanced use of natural resources while preserving them to meet the needs of the present without compromising the ability of future generations to do the same. This concept is grounded in fundamental principles that inform practice and policy across various fields, including economics, health, and environmental law. These three areas are often referred to as the “tripod of sustainability” (14).

The Triple Bottom Line (TBL) approach considers the three dimensions of sustainability: social, economic, and environmental. This approach is fundamental to assessing the impact of business practices and policies, ensuring that decisions consider not only profit but also social wellbeing and planetary health. These principles ensure that human actions respect the limits of ecosystems (15).

A key document on this topic is the Brundtland Report, which defines sustainable development as development that “meets the needs of the present without compromising the ability of future generations to meet their own needs” (16).

One of the fundamental principles of sustainability is intergenerational equity, which emphasizes the need to ensure that future generations have access to the same resources and opportunities as those available to the current generation. This principle is often linked to social justice and the conservation of natural resources, reflecting the need for the responsible and equitable use of available resources (17).

Another essential principle is precaution, which holds that uncertainty should not be used to delay measures that could prevent environmental damage in the absence of scientific consensus. This principle is widely discussed in environmental law, where the aim is to avoid ecological degradation before it occurs rather than remedy damage afterwards. The polluter-pays principle is also crucial, as it establishes that those who cause pollution must bear the associated costs of remediation, thereby promoting greater environmental responsibility (17).

Green chemistry is also a vital component of environmental sustainability. It aims to minimize the environmental impact of chemical processes by encouraging the use of safe and sustainable methods, thereby aligning with the principles of sustainable development. The concept is based on twelve principles that guide chemical practice to promote environmental sustainability. These include atom economy, which aims to maximize the incorporation of all materials used in the final synthesis, and minimizing solvent use by promoting reactions that can occur under solvent-free conditions (18). Like Green Chemistry, Green Pharmacy emphasizes the materials involved and the associated processes, including drug formulation and production. Both ideas aim to develop new methods to reduce waste, save energy, and identify alternatives for harmful materials, including solvents, raw materials, or final products. This applies to active pharmaceutical ingredients (APIs), as well as additives and packaging (19).

These technical approaches are consistent with a broader, systemic understanding of sustainability as articulated through the SDGs, which frame health and pharmaceutical activity within interconnected environmental, social, and economic systems. Embedding such principles into professional education supports the development of competencies in systems thinking, ethical responsibility, and anticipatory decision-making (20). At the institutional level, the integration of SDG-oriented sustainability strategies within higher education, as exemplified by the Belgian example of structured SDG implementation at the University of Namur (UNamur), illustrates how these global frameworks can be operationalized in academic contexts aligned with sustainability-oriented training objectives (21). In this regard, professional organizations such as the International Pharmaceutical Federation (FIP) have also emphasized, since 2016, the central role of pharmacists and their associations in leading efforts to address the environmental challenges associated with human and veterinary medicines and pharmaceutical practice (22).

Clearly, understanding environmental sustainability involves more than just preserving natural resources; it also requires a commitment to reducing the negative ecological impacts of human activities, such as pollution, resource waste, and ecosystem degradation (23).

In contemporary discussions of development, sustainability has become a central concept, reflecting the need to balance economic growth, social justice, and environmental protection. In the current context of global challenges, including climate change, environmental degradation, and social inequality, adopting sustainable practices is essential to ensure a viable future for generations to come. One of the most critical aspects of sustainability is corporate environmental responsibility. Companies play a vital role in promoting sustainable development, as their practices directly impact the achievement of global environmental goals (24).

Education plays a decisive role in promoting sustainability. Curricula must address their multiple dimensions to prepare students for the challenges of the 21st century. In this context, Education 4.0 emerges as an innovative pedagogical model aligned with the Fourth Industrial Revolution. It integrates digital technologies, including artificial intelligence, robotics, and big data. It emphasizes critical thinking, complex problem-solving, creativity, collaboration, and digital literacy—all essential skills for addressing contemporary environmental challenges (25). Education 4.0 also promotes problem-based learning and transdisciplinary projects that link sectors such as economics, ecology, health, and technology. This allows students to develop innovative solutions to real-world issues, such as water scarcity or pollution, through design thinking and sustainable entrepreneurship. Thus, science education is shifting toward a paradigm in which training is used not only to impart knowledge but also to empower professionals to effectively, critically, and ethically intervene in the transition to greener societies. In this sense, fostering a culture of sustainability within higher education institutions is an ethical imperative and an essential strategy for ensuring that future agents of change can implement sustainable practices within their communities and organizations (26).

Transforming consumption practices is also key to achieving sustainability. The concept of sustainable consumption—making conscious choices about products and services that minimize environmental impact—is becoming increasingly important. Events and initiatives that encourage responsible consumption behaviors can be an effective way to engage the community and raise awareness of the importance of sustainability. Collaboration among sectors of society, including governments, businesses, and non-governmental organizations, is crucial for promoting sustainable consumption practices (27).

The intersection between technology and sustainability is also essential. Digitalization and technological innovation present significant opportunities to enhance resource efficiency and mitigate environmental impact. The transition to renewable energy sources, for example, is an area in which innovation plays a crucial role, as does investment in technologies that optimize resource use and reduce carbon emissions (27). Innovative solutions are also needed in the pharmaceutical industry, where sustainable manufacturing practices and advanced research approaches can reduce environmental impact while meeting the growing demand for safe, effective, and affordable medications (28).

To operationalize and monitor environmental performance at individual, organizational, and sectoral levels, environmental impact indicators such as the ecological, carbon, and water footprints have emerged (29).

The ecological footprint is a measure of the amount of biologically productive land and sea area required to support a population's consumption of resources and absorb its waste, reflecting the pressure that human activity puts on ecosystems. It enables the demand placed on the planet by humans to be compared with its regenerative capacity and is widely used to assess the sustainability of countries, cities, or activities. The carbon footprint represents the amount of greenhouse gas (GHG) emissions associated with a human activity or product that contributes to climate change. However, there is no consensus on how to measure or quantify the carbon footprint. Some authors argue that it should measure only total carbon dioxide (CO_2_) emissions, while others stress the importance of accounting for all greenhouse gases (GHGs) directly or indirectly associated with an activity. To enable comparison across different gases, emissions are often expressed in carbon dioxide equivalents (CO_2_e), i.e., in units that reflect the global warming potential of greenhouse gases (30). The water footprint assesses the volume of freshwater used and polluted throughout the life cycle of a product or process, highlighting the industry's impact on global water resources and its sustainability implications (31).

The healthcare sector is a major contributor to GHG emissions. The health care sector is responsible for 4.6% of global GHG emissions, and pharmaceuticals are among the most significant contributors within this sector, accounting for 10%−55% of total health care GHG emissions (32, 33). This is mainly due to factors such as manufacturing processes, energy consumption, and the carbon footprint of pharmaceutical products and their disposal. Both GHG emissions and pharmaceutical pollution resulting from the provision of pharmacy services have a direct impact on health (34).

### 2.1 Occurrence of pharmaceutical residues worldwide

The intensive use of healthcare products, inefficient treatment at wastewater treatment plants (WWTPs), and the disposal of untreated wastewater have contaminated water bodies with these substances. Rivers are the preferred receiving environment for these effluents and are therefore focal points for their presence, particularly APIs and their metabolites (35).

A global study assessed pharmaceutical pollution in 258 rivers worldwide, representing 471.4 million people in 137 geographical regions. Samples were collected from 1,052 locations in 104 countries, spanning all continents. The highest cumulative concentrations of APIs were observed in sub-Saharan Africa, South Asia, and South America. The most contaminated sites were found in low- and middle-income countries, particularly in areas with poor wastewater, waste, and pharmaceutical management infrastructure (36).

This study observed the highest concentrations of APIs in water resources receiving effluents from the pharmaceutical industry (e.g., Bangladesh) or in environments receiving untreated effluents (e.g., Tunisia) (36).

Undoubtedly, the pharmaceutical industry is a significant source of environmental contamination, particularly when wastewater is not treated correctly (37).

Compounds such as synthetic hormones (e.g., 17α-ethinylestradiol), anti-inflammatory agents (diclofenac, ibuprofen), antiepileptics (carbamazepine), antidepressants, and beta blockers have been identified in surface water, groundwater, and even drinking water, even after passing through secondary wastewater treatment plants. The persistence of these compounds is attributed to their high chemical stability and resistance to conventional treatment techniques, and they are often detected at ng/L concentrations in aquatic environments (38–41).

Several studies have shown that effluents from this industry contain, among other APIs, high concentrations of antibiotics, creating selective pressure that favors the emergence of resistant bacteria (RB). The absence of regulations limiting the discharge of these compounds exacerbates the problem. To mitigate this impact, it is crucial to implement treatment technologies, such as specialized bioreactors and advanced oxidation processes (including UV, ozone, and plasma oxidation), before discharge. Pharmaceutical waste, from industry, hospitals, and domestic use, includes expired or unused tablets, capsules, ampoules, creams, and solutions. When disposed of in landfills without proper treatment, they generate leachates that can contaminate soils and aquifers with bioactive compounds. Unsealed landfills become anoxic environments that facilitate the horizontal transfer of resistance genes. Disposing of pharmaceutical waste in conventional landfills is a severe environmental failure. Effective alternatives include incinerating the waste at temperatures above 1,000 °C (which requires subsequent ash treatment) or using controlled digestion with UV radiation or ozone to degrade the drugs (37).

Microplastics and sludge from WWTPs act as vectors for antibiotics, resistance genes (RGs), and resistant bacteria (RBs) due to their high surface area, which facilitates the adsorption of these compounds and biofilm formation, thereby enhancing gene exchange. Sludge, often used as fertilizer, contains residues of RBs and RGs that can enter soil and water. Conventional landfills accumulate chemical residues and resistant microorganisms, creating reservoirs of resistance under selective pressure from antibiotics, heavy metals, and nutrients. Leachates from these landfills have been confirmed to contain RGs, RBs, and pharmaceuticals, indicating their potential for environmental dissemination. Antimicrobial resistance (AMR) is a major global health threat, with the WHO estimating 5 million deaths annually linked to resistant infections, exacerbated by environmental pollution. Hospital and industrial wastewater significantly influence microbial gene expression, altering natural microbiota and promoting multi-resistant pathogens. Studies show that *Escherichia coli* exposed to microplastic biofilms exhibits increased resistance compared to conventional settings, as microplastics support the formation of robust biofilms that shield bacteria from antibiotics (42, 43).

To address the issue of pharmaceuticals and antimicrobial resistance (AMR) in wastewater discharges, an integrated approach is necessary. This includes systematic monitoring and technologies like ozonation, UV radiation, and ultrafiltration, which effectively remove micropollutants and reduce resistant microbial loads. Strengthening legislation with discharge limits for pharmaceuticals and requiring advanced treatments in WWTPs is crucial. Additionally, research should investigate the interactions among pharmaceuticals, biofilms, microplastics, and microbial ecosystems to enhance ecological risk assessment and mitigate AMR (44).

Although APIs and metabolites have been included on the European Union's list of priority substances under the Water Framework Directive (45), many of them remain unregulated and are not regularly monitored. Studies recommend an integrated approach that incorporates advanced remediation technologies and extensive monitoring and assessment using reactive, advective, and dispersive transport models adjusted to different geographic regions (46).

Directive 2013/39/EU first recognized contamination of water and soil by pharmaceutical waste as an environmental issue (45). To monitor emerging pollutants and build a reliable database for identifying priority substances, the Directive created a watch list system, limited to 10 substances or groups, with specified monitoring matrices and cost-effective analytical methods. The first watch list (Decision 2015/495/EU) included seven APIs: two natural hormones (estrone, E1; 17β-estradiol, E2), one synthetic hormone (17α-ethinylestradiol, EE2), diclofenac (an NSAID—non-steroidal anti-inflammatory drugs), and three macrolide antibiotics (erythromycin, clarithromycin, and azithromycin) (47). This was updated by Decision 2018/840/EU, which removed diclofenac (due to sufficient monitoring data) and added amoxicillin and ciprofloxacin, supporting the EU's One Health Action Plan on Antimicrobial Resistance (48). The latest list (Decision 2022/1307/EU) includes nine APIs and metabolites: sulfamethoxazole, trimethoprim, venlafaxine and O-desmethylvenlafaxine, clotrimazole, fluconazole, miconazole, clindamycin, ofloxacin, metformin, and guanylurea (49).

### 2.2 Environmental impacts on health

Although medicines, including both human and veterinary medicinal products, exist to improve the health and wellbeing of billions of people and animals worldwide, their production and use can harm ecosystems and human health if appropriate measures are not taken to manage these potential environmental impacts, particularly aquatic contamination. Veterinary medicines, used on a large scale in livestock production and companion animal care, represent a significant pathway for pharmaceutical emissions into the environment. Recognizing these risks, international organizations have emphasized the importance of sustainability within health systems. According to the World Health Organization (WHO), health systems should adopt more sustainable practices to reduce their ecological footprint, given that they consume significant resources and generate substantial waste. The report emphasizes that environmental sustainability should be prioritized in all areas, including health systems, which should integrate innovative solutions to mitigate environmental impacts without compromising service quality (50).

Furthermore, the relationship between the environment and human health is well-established, with studies demonstrating that environmental factors can significantly influence the incidence and prevalence of various diseases. The interconnections between the quality of natural resources and social determinants of health have been identified, with factors such as air pollution, water contamination, and ecosystem degradation disproportionately affecting vulnerable populations. Therefore, several initiatives have been proposed to address these issues, emphasizing the need for an integrated approach, such as the “One Health” perspective, which recognizes the interconnectedness of human, animal, and environmental health (51).

Among the various environmental factors influencing human health, water quality stands out as a crucial determinant, directly linking ecological degradation to the spread of diseases and public health risks. This can cause a range of diseases, from gastrointestinal infections to chronic complications (51, 52). Water pollution resulting from chemicals and pathogenic organisms leads to a deterioration in public health and an increase in communicable diseases. Sources of contamination include industrial and domestic effluents, as well as intensive agriculture, which uses fertilizers and pesticides and is responsible for degrading aquatic ecosystems. For example, the presence of fecal coliforms in natural waters is directly related to outbreaks of intestinal diseases, such as diarrhea, which is one of the leading causes of infant mortality in developing countries (53). Furthermore, poor water quality can directly impact agricultural production and food security. Contaminated water used for irrigation can lead to the bioaccumulation of contaminants in farm products, which can enter the food chain and have harmful effects on ecosystems and consumer health. Inadequate management of water resources can exacerbate these problems, particularly in areas with poor infrastructure where monitoring and treatment of water and sewage are limited. Therefore, implementing effective treatment systems and continuous water quality monitoring strategies is crucial for protecting public health and ensuring the availability of safe drinking water (38, 54).

Several of these water contaminants belong to the group of so-called contaminants of emerging concern (CECs) (55). The CECs encompass numerous micropollutants, including specific pesticides and their degradation or transformation products, industrial chemicals (such as surfactants and their residues, gasoline additives, brominated flame retardants, plasticizers, and perfluorinated compounds), disinfection by-products, personal care products (PCP), human and veterinary pharmaceuticals and their metabolites, as well as nanomaterials, among others. Given the variety of structures, physical-chemical, and biological characteristics, particular focus has been placed on active pharmaceutical ingredients (APIs), which are unlike traditional pollutants, as they are purposefully engineered to exert biological effects, even at trace concentrations (56).

The impact of chronic exposure to these contaminants on humans, animals, vegetation, and aquatic species is a matter of concern for the scientific community and regulatory and supervisory entities, since they may not only compromise the functioning and maintenance of ecosystems, which is crucial to promoting the ecosystem services on which humanity depends, but also to cause various public health problems (57, 58).

Emerging human-health concerns arise via two principal routes: (i) endocrine disruption, and (ii) antimicrobials driving antimicrobial resistance (AMR) through environmental reservoirs—a documented pathway to human morbidity and mortality, now emphasized in UNEP/WHO quadripartite guidance (59).

The dissemination of AMR across environmental compartments occurs via three principal mechanisms. The first involves the environmental propagation of pathogenic microorganisms through contaminated water, soil, and animal manure, thereby potentially increasing the incidence of infections that necessitate antibiotic intervention. The second mechanism involves the covert transmission of low-inherent-pathogenicity resistant microorganisms to more susceptible populations, thereby amplifying the impact of resistance. For instance, approximately 14% of the global population harbors *Escherichia coli* in their feces that produce broad-spectrum β-lactamases, conferring resistance to antibiotics such as penicillins, cephalosporins, and, in some cases, carbapenems. Finally, excretions from human and veterinary use, along with the disposal of unused or expired antibiotics and effluents from pharmaceutical manufacturing, can further promote resistance by generating environmental conditions conducive to the horizontal transfer or emergence of novel resistance genes (60–62).

Estrogenic hormones, including E1, E2, and EE2 are recognized endocrine disruptors that affect aquatic organisms and human health at low concentrations (63). The EE2 used in oral contraceptives has strong ecological evidence of endocrine disruption: feminization and intersex in wild fish downstream of wastewater outlets, reductions in biomass, and altered foraging that propagate through pelagic food webs. Experimental and mechanistic work (including transgenic zebrafish) demonstrates windows of heightened sensitivity and exposure-history effects, supporting biological plausibility for developmental vulnerability in vertebrates (64, 65).

Regarding water policy (66), EE2 is prioritized not because it is more relevant to human exposure, but because it is non-physiological, highly potent, and less buffered by endocrine feedback mechanisms (67). Regarding hormone-related cancers, EE2 is classified as carcinogenic based on pharmaceutical-dose exposure, with associations to breast cancer, endometrial cancer, and ovarian cancer (68). However, to date, epidemiologic evidence relates to therapeutic or contraceptive use, not low-level environmental exposure. No human disease or condition has been epidemiologically attributed to EE2 exposure from drinking water; risk assessment relies on toxicology and conservative modeling rather than on population-level evidence, and ongoing concerns relate to cumulative estrogenicity and mixtures, not to EE2 alone. Therefore, while human epidemiologic attribution, for example, to hormone-dependent cancers from low-level drinking-water EE2 remains inconclusive, precautionary governance and improved surveillance are warranted considering cumulative mixtures and sensitive populations (69, 70).

Air quality is also a key factor in public health, as it directly impacts respiratory and cardiovascular diseases. Air pollution, primarily generated by the combustion of fossil fuels, industrial emissions, and vehicle traffic, results in the emission of fine particles (PM2.5 and PM10), nitrogen oxides, sulfur dioxide, and volatile organic compounds (VOCs). These substances are extremely harmful to human health. Prolonged exposure to high concentrations of these pollutants can lead to chronic respiratory diseases such as asthma, chronic bronchitis, and chronic obstructive pulmonary disease (COPD), and is also linked to an increased risk of death from cardiovascular disease (71).

Beyond its direct effects on health, air pollution exacerbates climate change, which may indirectly affect public health. Rising air pollutants due to climate change cause severe weather events like heatwaves and heavy storms, which can worsen health issues, especially in at-risk groups, including children and the elderly. Consequently, the investigation and formulation of public policies to reduce air pollution are crucial for safeguarding public health and promoting a healthy environment (72).

The increasing frequency and intensity of climate-related extreme events not only aggravate environmental contamination, including water pollution, but also expose communities to immediate and long-term public health consequences. These include threats to food security, the proliferation of infectious diseases, and disruptions in access to essential healthcare services. The capacity of communities to respond to such crises is often constrained by socio-economic factors, such as limited resources and infrastructure, which further exacerbate vulnerability. Addressing these challenges requires integrated adaptation strategies that simultaneously consider public health, environmental protection, and infrastructure resilience. Initiatives promoting sustainable land management, resilient construction practices, and environmental literacy are therefore essential to strengthening community preparedness and mitigating the health impacts of climate change (73).

In this context, health and pharmaceutical education play a critical role, as future professionals must be equipped to understand and respond to the complex interactions between climate, environment, and health. Engagement with broader educational initiatives, such as the European Network for Climate Change Education, positions pharmaceutical training within a European ecosystem that promotes climate and sustainability literacy across disciplines (74). Such initiatives reinforce the systemic nature of curriculum greening and support the development of competencies necessary to address climate-related health risks in a coordinated and anticipatory manner.

Accordingly, universities are key drivers of sustainability transitions through education and innovation. Integrating environmental sustainability into pharmaceutical curricula is thus essential to addressing the health challenges posed by climate change (75).

## 3 The role of pharmacists in environmental preservation

Although pharmacists are historically linked to administering medications and providing direct patient care, they have emerged as major advocates of behaviors aimed at reducing negative environmental impacts. They've also incorporated ecological health into their responsibilities and educated colleagues and the public about environmental challenges. As a result, regardless of pharmaceutical activity, the involvement of numerous pharmaceutical sectors and professionals is critical.

### 3.1 Drug's life cycle

**Figure 1:**
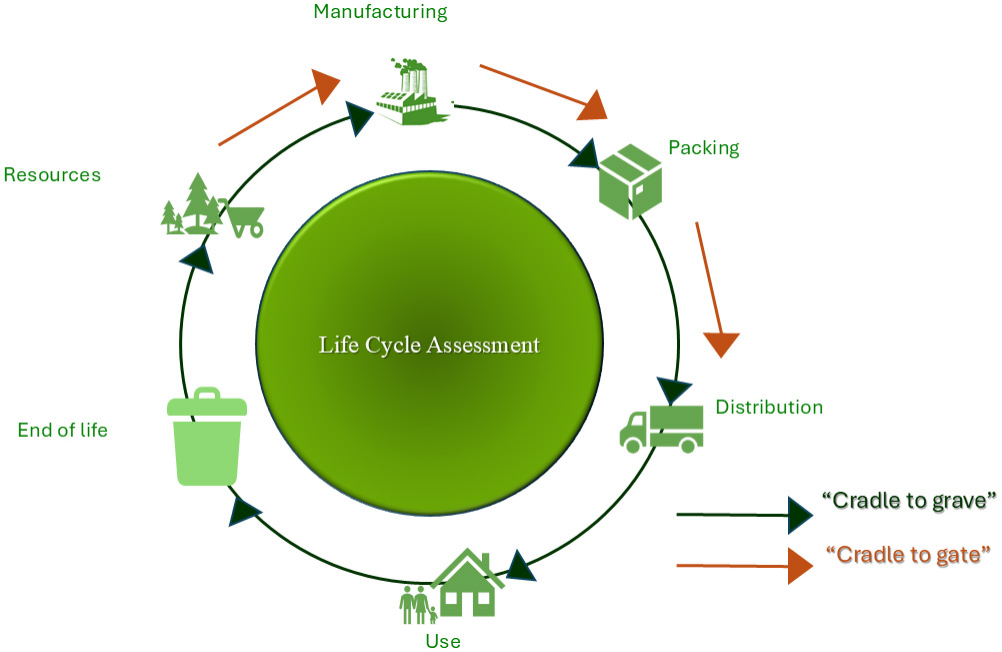
Life cycle assessment approaches.

The life cycle of a drug includes stages such as raw material acquisition, production, distribution, consumption, and disposal, all of which can generate waste that harms ecosystems. Life Cycle Assessments (LCA) are used to measure the environmental impacts throughout the life cycle. Initially, LCA focused on a “cradle-to-gate” approach, covering the entire production process. However, there is now a shift toward “cradle-to-grave” assessments, which account for the effects of distribution, consumption, and disposal, providing a comprehensive evaluation of the entire life cycle's environmental impact (76). Figure 1 presents these two different approaches in the context of LCA.

In a general LCA, three phases are recognized: upstream, central, and downstream. The upstream phase involves the extraction of raw materials, their supply to production, and precursor synthesis, with many pharmaceutical companies sourcing materials predominantly from Asia. This practice often overlooks suppliers' emissions, resulting in an underestimation of environmental costs. To address these issues, pharmaceutical companies should incorporate supply chain phases into their LCA and select suppliers that adhere to green chemistry principles to reduce greenhouse gas emissions. The central phase encompasses production and packaging, while the downstream phase includes distribution, consumption, and disposal, as well as analyzing potential environmental impacts from drug releases (77).

The integration of LCA into the pharmaceutical curriculum enables students to develop a comprehensive understanding of the environmental burdens associated with drug production and healthcare systems, guiding the creation of more sustainable pharmaceutical products and practices. These concepts can be integrated across disciplines such as pharmaceutical technology, chemistry, pharmacognosy, pharmaceutics, pharmaceutical engineering, social and administrative pharmacy, and pharmacoeconomics, fostering a holistic understanding of the environmental impacts associated with drug discovery, development, manufacturing, regulation, and use—empowering future professionals to design and implement more sustainable pharmaceutical products and systems.

### 3.2 Deprescribing

Deprescribing is the process of intentionally stopping a medication or reducing its dose to improve the person's health or reduce the risk of adverse side effects (78). It is a hot topic, with international interest driving new policy across the globe. Deprescribing represents a powerful yet often underexplored lever for sustainable pharmaceutical practice, as it reduces unnecessary medication use, minimizes environmental burden, and reinforces ethical and patient-centered care (79). Educational platforms such as deprescribe.org provide structured, evidence-based resources to support the integration of deprescribing into pharmaceutical training (80).

### 3.3 Pharmacists in the city (community practice)

Community pharmacists can mitigate pharmaceutical emissions by embedding environmental stewardship within medication reviews, deprescribing consultations, and patient education. Evidence-based deprescribing of long-term therapies (e.g., proton-pump inhibitors, benzodiazepines, chronic non-steroidal anti-inflammatory drugs) reduces unnecessary consumption and downstream excretion, with co-benefits for safety and adherence (81, 82).

Given their community presence and frequent role as the initial point of contact for healthcare, pharmacists are ideally positioned to spearhead environmental awareness initiatives. These initiatives can include drug recycling campaigns, guidance on environmentally friendly disposal of pharmaceutical products, and raising awareness of the responsible use of natural resources (83). This meets the need for environmental education and strengthens community confidence in pharmacists as health professionals committed to global wellbeing. Pharmacies can therefore become information and resource centers for the local population, providing information on the environmental impact of medicines and offering advice on reducing waste and promoting sustainable alternatives, such as lower-toxic medicines and recyclable packaging (84). Therefore, pharmacists can normalize responsible disposal, actively promoting medicine take-back schemes and discouraging sink/toilet disposal, which is known to reduce the number and amount of APIs entering wastewater systems (85, 86).

Pharmacies can also adopt a more environmentally responsible approach by implementing sustainable processes in their daily operations. For example, they can adopt Green Pharmacy principles by integrating environmental awareness into their operations, such as using non-toxic cleaning products, optimizing energy use, and contributing to more sustainable waste management (87). Additionally, they can consider using environmentally responsible packaging materials and identifying suppliers that prioritize sustainability in their production chain. Implementing technologies to manage stock and reduce waste can also contribute directly to environmental preservation in the pharmaceutical sector (83).

### 3.4 Pharmacists in clinical and hospital settings

In hospitals, clinical pharmacists operationalise environmental preservation through medicines optimisation, deprescribing, antimicrobial stewardship (AMS), medication reconciliation across transitions of care, and device-level therapeutic substitution, embedding sustainability into routine safety and quality processes. Pharmacist-led interventions improve prescribing quality, reduce medication-related harm and healthcare utilization, which, when aligned with respiratory device policy, yield meaningful carbon savings without compromising clinical outcomes. These actions translate indirectly into environmental benefits by lowering the volume of APIs excreted to wastewater, reducing upstream manufacturing emissions, and decreasing avoidable care episodes that carry additional resource and carbon burdens (81, 82, 88).

Pharmacists conduct structured medication reviews to identify potentially inappropriate medicines (PIMs), optimize treatment duration, and streamline regimens for older, multimorbid inpatients and outpatients (89).

A 2024 pragmatic clinical trial integrating pharmacist reconciliation at admission and discharge reduced clinically important discharge errors by ~20-fold compared with control wards, with counseling embedded in routine practice. Such reductions have sustainability co-benefits, such as less remedial care, re-prescribing, and transport, alongside direct safety gains (88).

Pharmacist-led AMS programmes consistently improve appropriateness and reduce antibiotic consumption, length of therapy, and costs, with signals for lower mortality. These stewardship gains directly link to environmental preservation by curbing antimicrobial loads in effluents and potentially slowing AMR-related resource use (90).

Within respiratory prescribing, pharmacists can lead programmes that prefer dry-powder inhalers (DPIs) to hydrofluoroalkane-propelled metered-dose inhalers (MDIs) when clinically appropriate, delivering major GHG savings. Broader respiratory sustainability analyses and guidance letters emphasize that optimizing disease control (reducing rescue short-acting beta-agonist overuse) further decreases the carbon burden of care pathways, again an arena where hospital pharmacists can align device/formulary policy with guideline-concordant therapy (91).

Qualitative work in National Health Service (NHS) hospitals indicates that many pharmacists would benefit from better access to environmental evidence, tools, and time to incorporate sustainability into day-to-day clinical decision-making. In parallel, national net-zero programmes increasingly foreground medicines optimisation and inhalers as priority levers, embedding pharmacy teams in green plans, carbon-footprinting initiatives, and integrated care checklists. Hospital pharmacy leadership can therefore link deprescribing, AMS, and reconciliation to organizational sustainability metrics and reporting frameworks, ensuring clinical quality actions contribute measurably to net-zero objectives (92).

### 3.5 Pharmaceutical industry

The pharmaceutical industry, as a vital sector of the economy and public health, faces growing criticism regarding its contribution to environmental pollution.

The production of medicines involves the intensive use of chemicals and various processes that can be inherently polluting. Pharmaceutical factories often release atmospheric and liquid pollutants containing volatile organic compounds (VOCs), which are frequently used as solvents or intermediates in the synthesis of drugs. Many of these VOCs are toxic and contribute to the formation of tropospheric ozone (photochemical smog). Examples include toluene, acetone, chloroform, methanol, ethanol, and dichloromethane. Other pollutants include chemical residues remaining after chemical reactions or drug purification. These residues include strong acids and bases, metal salts, residual peroxides, and contaminated washing solutions. By-products are undesirable secondary compounds formed during synthesis reactions or as a result of the decomposition of drugs. Examples include synthesis impurities, organochlorine by-products, and oxidative or photolytic degradation products. The emission of these pollutants can contribute to the formation of acid rain, which harms aquatic and terrestrial ecosystems (93).

One of the challenges faced by the pharmaceutical industry is managing by-products. Many of the intermediates and waste products used in synthesis processes are not adequately treated, resulting in the release of toxic compounds into the environment. These substances can be persistent and accumulate in soil and water, potentially affecting aquatic life and human health. Furthermore, industrial processes require significant amounts of water and energy, exacerbating the scarcity of natural resources in regions with limited water (94).

The pharmaceutical industry is one of the most energy-intensive and greenhouse gas-emitting sectors within the industrial landscape. This results from the complexity of manufacturing processes, the need for high product purity, strict environmental control at all stages of production (e.g., in clean rooms), and specialized logistics for storing and transporting medicines. These factors collectively contribute to a substantial carbon footprint, which is often underestimated due to the sector's social and health prestige. A study on the carbon footprint of the global pharmaceutical industry from 2006 to 2015 found that the world's largest pharmaceutical companies emitted approximately 48.55 million tons of CO_2_ equivalent per year. For comparison, this is approximately 13% higher than that of the automotive industry over the same period, despite the latter producing many more units and having greater public visibility in the debate on energy transition (95). The main elements responsible for these emissions in the pharmaceutical industry are:

i. Chemical synthesis of drugs. Many synthetic routes involve multiple reaction steps, the use of organic solvents, energy-intensive purification processes, and sensitive reagents. It is estimated that drug synthesis accounts for 20%−30% of a pharmaceutical company's total carbon footprint (96).
ii. Energy use in manufacturing facilities: factories often operate 24 h a day and have strict temperature and humidity controls, particularly in sterile production facilities. These facilities require highly energy-intensive heating, ventilation, and air conditioning systems (97).
iii. Indirect energy consumption: this includes the production of raw materials and packaging, particularly plastics, as well as continuous refrigeration in the cold chain and specialized transport (98).
iv. Globalized distribution: dependence on international supply chains, whereby APIs are produced in Asian countries and finished products are distributed worldwide, involves extensive use of air and sea transport, both of which emit a lot of carbon (99).

In recent years, several companies and industrial consortia have committed to reducing their GHG emissions to align with the objectives of the Paris Agreement and the EU's goal of achieving carbon neutrality by 2050. Some of the strategies adopted include: (i) replacing fossil fuels with renewable energies, such as solar or wind power, in production units (e.g., Roche and Novartis already partially operate with green energy); (ii) optimizing synthesis processes by applying green chemistry principles, reducing the number of steps, and using fewer toxic solvents; (iii) conducting life cycle assessments (LCAs) to identify critical points of environmental impact throughout the value chain; and (iv) designing more sustainable packaging and reducing single-use materials (100).

Although not as evident as in other fields, the pharmaceutical sector's carbon footprint is substantial and requires immediate action. Shifting toward low-carbon manufacturing practices necessitates creativity, suitable regulations, and financial incentives. It's essential to minimize environmental impacts without compromising the effectiveness of medications. This should be established as a key strategic focus in an industry facing growing demands to show accountability in social and environmental matters.

The pharmaceutical industry also has a significant water footprint due to the large volumes of water required for drug manufacturing, cleaning, and cooling processes. Reducing this footprint is becoming increasingly important as companies strive to enhance sustainability through water recycling, efficient purification, and advanced wastewater treatment technologies (101).

In conclusion, industrial pharmacists can embed environmental performance in product design and manufacturing through LCA, greener synthesis routes, solvent minimization, and process intensification. Integrating environmental endpoints (persistence, bioaccumulation, and toxicity) during formulation and scale-up supports a lower overall footprint and facilitates downstream regulatory acceptance (102, 103).

### 3.6 Packaging and distribution logistics

The production and improper disposal of packaging, alongside the logistical processes involved in distributing pharmaceutical products, significantly impact the environment.

While medicine packaging is crucial for protecting the product during its shelf life, it is often made from non-biodegradable materials such as plastic and aluminum. Many of the polymers used for this purpose are not biodegradable and can take hundreds of years to decompose, resulting in waste accumulating in landfills and the natural environment (104).

Plastic, a primary component of pharmaceutical packaging, possesses characteristics that render it resistant to degradation and significantly contribute to both marine and terrestrial pollution. Furthermore, producing this packaging contributes significantly to carbon emissions, as manufacturing plastics and aluminum requires high energy consumption and releases greenhouse gases (GHGs), thereby contributing to climate change (105).

Analysis of the life cycle of pharmaceutical packaging reveals that each stage, from the extraction of raw materials to their end use, is associated with environmental impacts that affect public health and the ecosystem. Although reuse and recycling can minimize these effects, the recycling rate for pharmaceutical packaging remains low. This is partly due to a lack of awareness among consumers and healthcare professionals regarding proper disposal methods. The growing movement toward sustainable and recyclable materials highlights the urgent need for innovation in medicine packaging. Adopting biodegradable materials, such as bioplastics or recycled paper, can reduce the environmental impact of packaging in the supply chain (6).

The distribution of pharmaceutical products involves several stages, from manufacturing to final delivery to consumers. However, this supply chain often generates significant waste. The transportation of medicines frequently requires excessive, non-recyclable packaging, contributing to increased plastic pollution. Furthermore, the distribution system typically relies on refrigerated transport, which is energy-intensive and generates additional carbon emissions from the use of fossil fuels. Maintaining a controlled temperature for sensitive medicines requires specific single-use packaging, which increases waste volumes (106). Reverse logistics also generate waste, as medicines are returned to the supplier due to recalls or short expiry dates. This additional waste is unregulated and requires careful management to avoid environmental contamination (107).

In logistics, improving the efficiency of transport and distribution is paramount. Implementing innovative technologies for tracking and route optimisation can help to achieve this. This involves optimizing routes to reduce costs and carbon emissions, as well as maximizing vehicle capacity to minimize the need for additional transportation.

Therefore, the transition to a circular economy model in the pharmaceutical industry is advantageous, as it needs a systemic redesign that extends beyond one-off recycling and encompasses the entire value chain—from packaging design to post-consumer collection. The “reduce, reuse, recycle” principles must be strategically integrated into the eco-design of pharmaceutical packaging by minimizing the number of components and materials used, removing non-recyclable elements (such as metallised films), and facilitating disassembly for recycling. Industrial trials have demonstrated that adopting single-material packaging (e.g., polypropylene blister packs) can significantly enhance recyclability while ensuring product stability (108).

The transition to more sustainable logistics solutions in the pharmaceutical industry has been driven by the adoption of reusable containers, inspired by studies in related sectors such as food distribution. Using reusable isothermal containers, such as boxes with vacuum insulation panels, offers significant environmental benefits compared to disposable packaging, such as expanded polystyrene (EPS). Even accounting for sanitation and return transport, VIP containers reused for 300 cycles achieved an 87% reduction in greenhouse gas emissions compared to disposable EPS containers. The study also shows that VIP packaging outperforms disposable alternatives in terms of carbon footprint after only seven reuse cycles, making it a highly effective solution for closed logistics systems such as hospitals. However, such systems require a high degree of digitisation for traceability, as well as adequate infrastructure for cleaning, repair, and end-of-life management (109).

### 3.7 Pharmacists in the regulatory field

Regulatory pharmacists are crucial in environmental risk assessments (ERAs) for medicinal products by analyzing predicted environmental concentrations (PECs), assessing potential endocrine-disrupting properties, and developing mitigation strategies such as risk management plans and labeling (67, 103). Moreover, they convert intricate ERA results into practical recommendations for national agencies and purchasing policies. Current methods involve adopting environmental impact classification systems, such as Sweden's publicly available environmental ratings for pharmaceuticals, which guide prescribers and payers and enable widespread substitutions (102).

### 3.8 Pharmacists in research

Research pharmacists contribute to green design paradigms, biodegradable APIs, and benign-by-design excipients, while working with engineers to enhance API removal in wastewater treatment (85, 86). They can bridge environmental exposure models with utilization data, generating decision-support tools that quantify environmental co-benefits of deprescribing, substitution, and procurement choices.

The pharmacists also work in various analytical laboratories, including clinical, toxicological, and research laboratories (biology and chemistry). In their activities, they can contribute to sustainability by ensuring the safe management and disposal of chemicals and minimizing environmental contamination. Their role in developing eco-friendly analytical methods and promoting efficient resource use supports sustainable laboratory practices and environmental protection (110).

### 3.9 Cross-cutting responsibilities of pharmacists

In various contexts, three overarching responsibilities deserve clear acknowledgment. Initially, deprescribing demonstrates quantifiable environmental advantages by lowering API discharge and waste (81, 111). Secondly, switching to lower-impact alternatives, such as using DPIs when clinically suitable, can lead to considerable reductions in carbon emissions and possible reductions in downstream ecotoxicity (91). Third, environmental impact scores for medications, based on ERA and LCA principles, can be created and shared by industrial and regulatory pharmacists to inform guideline committees, formulary groups, and national/European authorities (102, 103). Collectively, these positions implement environmental conservation while maintaining the priority of clinical effectiveness, safety, and equity.

## 4 Pharmaceutical curriculum and sustainability

### 4.1 Professional profile of pharmacists

The professional profile of pharmacists is complex and diverse, reflecting the evolution of the profession over time. As the public's appreciation of pharmacists' role in public health grows, it becomes increasingly important to understand the scope of their duties, the skills they require, the areas in which they work, and the challenges they face.

While pharmacists are traditionally viewed as specialists in medication responsible for the safe dispensing of medicines, their role extends far beyond this. They play a multifaceted role, with responsibilities that vary by workplace. These workplaces may include community and hospital pharmacies, the pharmaceutical industry, clinical analysis laboratories, bromatology and toxicology laboratories, research laboratories, regulatory affairs, and public health and education institutions (112).

Key responsibilities include advising patients on the proper use of medicines, identifying potential drug interactions, managing pharmacovigilance programs, and contributing to the development of public health policies. Pharmacists must have a thorough understanding of the pharmacological profiles of medicines, including their indications, contraindications, and potential adverse effects. This knowledge enables pharmacists to play an active role in patients' health, promoting adherence to treatment and preventing complications arising from its misuse (113).

In addition to technical knowledge, strong interpersonal and communication skills are essential for pharmacists as they need to build effective relationships with patients and other healthcare professionals. Being able to listen to and educate patients about their health conditions and treatments is crucial in a multidisciplinary team environment where healthcare decisions are made jointly (114).

Pharmacists can work in various areas, each requiring a distinct set of skills and knowledge. Community and hospital pharmacies, for example, offer the most obvious opportunities for patient interaction. In this context, pharmacists are responsible for counseling services, medication management, health education, drug therapy, and clinical interventions (115).

In the pharmaceutical industry, pharmacists are involved in researching and developing new medicines and ensuring the quality and safety of the final product. This work requires proficiency in scientific research and laboratory techniques, as well as an understanding of the regulations that govern the pharmaceutical and medical device industries (116).

Pharmacists play a crucial role in public health by participating in immunization programmes, awareness campaigns, and disease prevention strategies. They provide essential information about medications and treatments, helping dispel common misconceptions. Additionally, pharmacists educate and advocate for sustainability, guiding future professionals and the public toward responsible pharmaceutical practices that benefit both human health and the environment.

### 4.2 Academic curriculum vs. sustainability

Considering current environmental challenges, the inclusion of environmental sustainability principles in pharmaceutical science education has gained prominence worldwide. International health and pharmacy organizations emphasize the role of pharmacists in reducing the environmental impact of medicines. For example, the WHO advocates training “climate-smart” health professionals who can strengthen health systems and act as agents of intersectoral solutions to the climate crisis. In 2016, the International Pharmaceutical Federation (FIP) published a policy statement on Green Pharmacy Practice, recognizing the global relevance of the environmental impact of medicines and recommending that pharmacy schools integrate the ecological effects of drugs into their curricula and teach “green” principles (117).

In line with this, the Pharmaceutical Group of the European Union (PGEU), representing European community pharmacists, advocates for the inclusion of environmental aspects in pharmacy student training and professional development programs as part of a “One Health” approach (118).

These international guidelines, combined with European Union initiatives such as the European Green Deal and the growing focus on planetary health, provide a favorable context for revising pharmaceutical science curricula to train professionals who are aware of the ecological impact of medicines throughout their entire life cycle (119).

In the educational sphere, different countries have adopted various strategies to incorporate environmental sustainability content into their pharmaceutical science curricula. These strategies include specific courses on drugs and the environment, modules incorporated into compulsory subjects, interdisciplinary projects, sustainable laboratory practices, extracurricular activities, and green professional certifications. The aim in all cases is to equip future pharmacists with skills such as pharmaceutical waste management, environmental risk assessment of medicines, the rational use of drugs from an ecological perspective, the application of the circular economy to pharmacy, and socio-environmental responsibility in healthcare (120).

These diverse national initiatives collectively reflect a broader global movement to embed sustainability principles in pharmaceutical education. This development is also supported by international frameworks, such as the United Nations Sustainable Development Goals (SDGs), particularly SDGs 12 (Responsible Consumption and Production), 13 (Climate Action), and 3 (Good Health and Wellbeing), which include targets to reduce environmental pollution. Consequently, universities are aligning their curricula with these goals to identify areas where they can make practical contributions. Additionally, the concept of Planetary Health has served as a valuable framework for integrating knowledge from various disciplines in a coherent and holistic manner (121).

### 4.3 Sustainability in European pharmacy curriculum

#### 4.3.1 Portugal

In Portugal, initiatives by educational institutions and guidelines issued by professional bodies have led to a growing concern with integrating sustainability into the pharmaceutical curriculum. The Portuguese Pharmaceutical Society (OF) has emphasized that the traditional concept of “rational use of medicines” must now include an environmental dimension. This involves encouraging good practices in medicine management and strengthening the ability to minimize and treat pharmaceutical waste. The OF stance underscores the expectation that pharmacists will be trained from the outset to consider the environmental impact at every stage, from selecting the medicine to its end-of-life cycle (122).

Although the Integrated Master's Degree in Pharmaceutical Sciences (MICF) has a basic structure defined by European directives (123), some higher education programmes already include environmental components in their curriculum.

In 2023, the University of Lisbon's Faculty of Pharmacy (FFUL) launched a postgraduate course in environmental risk and health, addressing ecological and digital transitions. This transdisciplinary program, designed for professionals and students, encompasses ecological toxicology, risk assessment, and public health. Participants assess the impacts of environmental contaminants, with a focus on endocrine disruptors, antibiotics, and bacterial resistance. The course covers the regulatory aspects of ecological risk for medicines, cosmetics, biocides, and pesticides, incorporating case studies to provide practical experience. It emphasizes One Health concepts and aligns with the EU Green Deal to minimize the pharmaceutical sector's ecological footprint (124).

The Faculty of Pharmacy at the University of Porto offers an optional course, “Ecotoxicology and the Environmental Impact of Medicines,” that provides students with knowledge of the environmental effects of pharmaceuticals. The course covers principles of ecotoxicology, pharmaceutical contamination, and the impact of medications on soil and water. Students analyze case studies and discuss waste management, developing skills in environmental risk assessment and advising on proper medicine disposal (FFUP, 2007).

Although both courses cover current topics in sustainability and pharmaceutical activity, they are designed for a small number of students and/or professionals, as they are elective modules (FFUL) or postgraduate programmes (FFUP). Consequently, there is a gap in the sustainability training of students in the field of pharmaceutical science. This gap also exists in the current directive regulating pharmaceutical science courses, which does not address environmental issues (123).

The University of Lisbon recently launched a Doctoral Programme in Planetary Health, addressing global challenges at the intersection of health, environmental, social, and political sciences. The programme involves the 18 Schools of the University of Lisbon, and the faculty comprises respected experts from various fields, all committed to fostering an academic environment conducive to high-quality, impactful research. The program equips students to address complex ecological and societal issues through a holistic approach. By focusing on action and change, it identifies problems at local, regional, and global levels related to planetary health. Students develop and test solutions collaboratively in labs or simulations. This results-driven curriculum integrates diverse knowledge to tackle planetary health challenges, with doctoral topics shaped by real-world issues in partnership with stakeholders who may hire graduates (125).

#### 4.3.2 Belgium

In Belgium, the UNamur has explicitly integrated the SDGs into its teaching and learning strategies in health-related programmes, including pharmacy-relevant education, thereby fostering systems thinking and responsibility for the societal and environmental impacts of healthcare. UNamur maintains a catalog of courses that integrate SDG content by faculty, including the Faculty of Medicine (which hosts pharmacy studies at UNamur). Pharmacy-relevant modules span clinical development, pharmacist–patient relationship, physiology, psychology, and clinical trials, creating multiple entry points to discuss sustainability, systems thinking, and responsible practice within health curricula. Pharmacy-relevant modules span clinical development, pharmacist–patient relationship, physiology, psychology, and clinical trials, creating multiple entry points to discuss sustainability, systems thinking, and responsible practice within health curricula (21).

Université catholique de Louvain (UCLouvain) coordinates the interuniversity certificate “Soins de santé durable, agir pour transformer,” aimed at health professionals, including pharmacists (126). This programme is designed to shift the healthcare paradigm by equipping participants with the competencies needed to integrate sustainability, planetary health, and ethics into professional practice (20).

#### 4.3.3 United Kingdom

In recent years, there has been a growing emphasis on integrating environmental sustainability into pharmacy courses in the United Kingdom (UK). This has been driven by recommendations from several institutions, including the Royal Pharmaceutical Society (RPS), as well as pioneering initiatives from some universities. One notable example is the Greening project in the MPharm (Master of Pharmacy) curriculum at the University of Huddersfield. In this programme, the teaching team has incorporated environmental sustainability principles throughout the curriculum, adopting a strategy of reformulating pharmaceutical topics from an explicit ecological perspective. This means that traditional issues such as pharmacotherapy, pharmaceutical chemistry, and clinical pharmacy now include discussions on environmental impact. For instance, students are encouraged to consider the ecological impact of drugs during pharmacology or therapeutics classes, exploring “greener” alternatives such as drugs with lower environmental persistence or less aggressive formulations, without compromising clinical efficacy. Huddersfield also enhanced training in compulsory subjects with extracurricular lectures on climate change and environmental health, as well as “green pharmacy” research projects. This approach equips graduates with knowledge and skills to evaluate the drug lifecycle and suggest sustainable improvements (121, 127, 128).

The British experience is not confined to Huddersfield. Several pharmacy schools in the UK have incorporated environmental content into their curriculum in various ways. Some universities have introduced courses focusing on planetary health and pharmacy. In contrast, others have included one or two compulsory seminars on climate and health in existing public health or social pharmacy courses. This integration aligns with national guidelines from the English NHS, such as those from the Sustainable Development Unit (SDU), as the British health system strives for carbon neutrality and requires professionals trained in sustainable practices.

The SDU was a British government agency whose aim was to embed the principles of sustainable development, social value, and broader determinants of health across the health and social care system in England. In July 2022, as part of the NHS's net zero plans, the SDU was replaced by the Greener NHS National Programme (127).

#### 4.3.4 Germany

Germany has been addressing sustainability in pharmaceutical education through planned structural reforms and continuous professional training. Unlike the more isolated approaches adopted in specific curricular units, Germany's approach stands out for its coordinated movement to review national training standards and the initiatives of pharmaceutical entities to create standardized teaching programmes on climate and health.

A key development is the Approbationsordnung für Apotheker (AAppO), the federal regulation that defines the minimum curriculum for pharmacy faculty. Since 2020, the Bundesapothekerkammer (BAK, the Federal Chamber of Pharmacists) has held roundtable discussions and established working groups to incorporate the topics of “drugs in the environment” and sustainability into the new national pharmacy curriculum. The consensus reached resulted in an inter-institutional position for the environmental theme to appear across the various disciplines of the Pharmaceutical Sciences course. The draft of the new AAppO, which is scheduled to come into force on 1 October 2025, incorporates the concept of “planetary health” into the programme to be taught. This means that future pharmacists in Germany will be required to receive training on the impact of medicines on ecosystems, the risks of pharmaceutical waste, and strategies for minimizing environmental damage across subjects such as pharmacology, pharmaceutical chemistry, and pharmaceutical technology. It is worth mentioning that revising the AAppO aligns with German ecological protection policies and the country's commitments to the European Union's “Pharmaceuticals in the Environment” Action Plan, thereby strengthening the connection between public guidelines and the academic curriculum (129).

While German pharmacy faculties are still adjusting their courses individually, specific actions are already being taken. Proposals have been made to create “Sustainable Pharmacy” courses at universities, supported by the German Federal Environment Agency (Umweltbundesamt), which would increase the production of teaching materials and specific research in this area. Additionally, professional journals such as Deutsche Apotheker Zeitung have been disseminating sustainability-related content, facilitating educational discussions between students and professionals (129).

#### 4.3.5 Finland

Finland is one of the pioneering countries in integrating sustainability into its pharmacy programs. In 2016, the University of Helsinki (Faculty of Pharmacy) implemented a comprehensive curriculum reform to incorporate environmental aspects into all courses whenever justified. This involved, for example, introducing practical work in which students, during pharmaceutical care classes, must address sustainability issues in patient guidance (as part of pharmaceutical advice, including recommendations for proper disposal and waste reduction). Another innovative exercise required students to recreate a registration dossier for a new medicine, including preparing an environmental risk assessment (ERA) as mandated by regulatory authorities. In addition, Helsinki has established an annual academic event called “Green Pharmacy,” embedded in an existing course, with lectures by professionals on topics such as drug emission routes in the environment, environmental risk assessment methods, environmentally conscious pharmaceutical production, rational use from an ecological perspective, and actions to reduce pharmaceutical waste. This Finnish model stands out in that, rather than a single isolated course, sustainability is a common thread throughout the training, consistently reinforcing the message (130, 131).

#### 4.3.6 Sweden

Pharmaceuticals in the environment were mentioned in the general learning objectives of Swedish pharmacy Universities, aligned with the Higher Education Ordinance; however, content related to the environment was only included in curricula for some courses, primarily those related to sustainable development. Sweden's pharmacy programs include some education about the environmental impacts of pharmaceuticals, but the curricula could be further developed. Among the general learning objectives derived from the Higher Education Ordinance on pharmacy Master of Science and Bachelor of Science program curricula in Sweden, only one pertained to environmental issues related to pharmaceuticals, specifically emphasizing their rational and optimal use. Furthermore, two universities established local learning objectives concerning the environmental impacts of pharmaceuticals: (1) sustainability in the processes of drug development, production, sale, and utilization within the master's program at the University of Gothenburg, and (2) sustainable development in pharmaceuticals across both pharmacy programs at Uppsala University (132).

### 4.4 United States of America

In the United States, although there are still no formal requirements for specific accreditation standards on environmental sustainability, several pharmacy schools have incorporated the topic on their own initiative, often motivated by climate urgency and broader academic movements (such as planetary health in health schools). The incorporation occurs in both compulsory PharmD (professional doctorate in Pharmacy) courses, elective courses, and extracurricular activities. A notable example is the University of California, San Francisco (UCSF) School of Pharmacy, which has introduced a compulsory component on climate and pharmaceutical health. This is a 3-h session within a 3rd-year health policy course, dedicated to climate change and sustainability. This session has been carefully designed, including preparatory readings and prior activities, followed by an interactive class with a panel of health professionals and small group discussions based on real cases. The content covers the interrelationships between climate change and health (e.g., effects of extreme events on the medicine supply chain, increase in infectious diseases, and the need for pharmacist preparedness), as well as the role of medicines in the environmental footprint of the health sector (90).

Small groups analyse cases such as “... how pharmacies can respond to a prolonged heatwave affecting patients on multiple medications...” or “strategies to reduce waste in mass vaccination campaigns,” promoting critical thinking and practical solutions. This integrative approach develops students' skills in analyzing health policies from an environmental perspective and collaborative skills by including multiple professions on the panel (pharmacists, doctors, managers) (90).

The University of Charleston School of Pharmacy has added a Pharmacists and the Environment class to its Public Health course. This class covers the impact of extreme weather on health and access to medicine, eco-friendly practices throughout the medicine lifecycle, the health sector's carbon footprint, and best practices for disposal. Students learn about sustainable drug design, manufacturing, green logistics, and the rational use of drugs. This prepares them to promote sustainability in pharmacy and highlights the pharmacist's role in community health and environmental education, including efforts to address risks associated with pharmaceutical waste. Additionally, several American schools offer optional courses on planetary health and sustainability in healthcare for pharmacy students. At UCSF, pharmacy students can take “Earth Health: Sustainability in Health Care,” a 10-week course focused on climate change and sustainability aligned with the UN Sustainable Development Goals. Guest speakers share their sustainability projects to inspire student leadership in health. Although optional, it enhances pharmacists' training in the subject (90, 101).

Student action is also driving curricular change in the US. One notable tool is the Planetary Health Report Card (PHRC), initially created by Stanford medical students, which evaluates schools in categories such as curriculum, research, community engagement, and ecological footprint (102). Recently, the PHRC was piloted at two US pharmacy schools, where the indicators were adapted for pharmacy use, and expansion plans are underway. This initiative encourages colleges to improve their curricula (to obtain better scores), promotes the formal inclusion of sustainability content, supports student initiatives (such as “Pharmacists for Climate” groups), and creates research opportunities in environmental pharmacy (4, 103).

### 4.5 Australia

Australia is incorporating environmental sustainability into pharmaceutical training, with Monash University implementing a 1-week module on the “environmental impact of medicines” in the fourth year of its Pharmacy course, as part of the discipline “Rational Use of Medicines.” The module examines how medicines impact the environment throughout their lifecycle, from green chemistry considerations in production to environmental risk assessment. Activities include debates on medical return protocols, comparisons of packaging and pharmaceutical forms, and analyses of environmental contamination cases. The module links this content to the subject of rational use, emphasizing the importance of sustainability in prescribing and dispensing today (121).

In addition to Monash, other Australian pharmacy schools are also beginning to follow a similar path, although they are still in their embryonic stages. Recent research reveals a gap between student awareness and the formal presence of green content in curricula (5).

Australian pharmacy schools are addressing a discrepancy between student awareness and the inclusion of green content in curricula. A 2023 study found that 94% of students agreed that the pharmaceutical profession has a responsibility to adopt sustainability initiatives; however, only 8.5% had encountered any curricular content on environmentally sustainable pharmaceutical practices. This suggests that students' knowledge came from external sources or personal interest. At the same time, 99% had already received information about sustainability through lectures, media, or events (5).

Aware of this need, Australian universities have sought strategies for gradually integrating these issues into their curricula. In addition to short modules in subjects, efforts are being made to create dedicated optional curriculum units. One example is the offering of a course entitled “Medicines and the Environment” in collaboration with Åbo Akademi University in Finland, which has been made available to Australian students through exchange programmes or international consortia. These subjects address not only scientific aspects (ecotoxicology, environmental pharmacovigilance), but also global and local waste management policies and environmental legislation related to medicines, providing participants with an intercultural overview (121).

Australia also participates in global planetary health movements. Some pharmacy schools have joined the Planetary Health Alliance project and use tools such as the Planetary Health Report Card, initially developed by medical students in the US, now adapted to evaluate pharmacy schools on “green” criteria. This has encouraged healthy competition and improvements in curricula as institutions strive to enhance their “green score” by incorporating more sustainable content and eco-friendly practices (121).

Australia is undergoing a curriculum transition, reflecting the growing demand from students for the inclusion of this topic. The trend is that, driven by evidence and international pressure, more Australian universities will formalize robust sustainability components in their curricula, consolidating skills aligned with global standards (WHO, FIP) and local peculiarities (such as the Australian goal of reducing emissions in health and the national commitment to the UN Sustainable Development Goals) (121).

### 4.6 Environmental awareness in pharmaceutical science undergraduate programmes

Environmental awareness is essential in training health professionals, especially in pharmaceutical sciences. It is an ethical and professional requirement that addresses global challenges. Training modern pharmacists in this area equips them to take on a responsible, transformative societal role (132).

The integration of environmental consciousness into the Pharmaceutical Sciences curriculum extends beyond mere knowledge acquisition; it also fosters critical thinking and ethical awareness throughout the entire academic program. This consciousness enables students to view their future professional roles as integral parts of an interconnected system, in which their decisions will impact not only the patient but also the broader social, economic, and ecological contexts surrounding that patient. Cultivating environmental awareness involves preparing pharmacists to adopt a comprehensive and inclusive perspective on their contributions to the healthcare system. Such preparation instills a sense of responsibility toward the community, anchored in a profound recognition of the interconnectedness of health, society, and the environment (133).

Environmental awareness fosters the development of cross-cutting skills essential for contemporary pharmaceutical practice. These skills include: (i) critical and systemic thinking, which involves relating complex variables and anticipating medium- and long-term consequences; (ii) informed decision-making, which involves weighing multiple dimensions—therapeutic, social, economic, and environmental—when making pharmaceutical choices; (iii) leadership and positive influence, which involves training professionals to promote good practices among colleagues, users and institutions; and (iv) communication and teaching skills, which involve raising awareness among users and communities about safe and responsible practices, thereby enabling pharmacists to take on an active educational role (117, 127).

Skills develop within specific contexts, deepening environmental awareness and enhancing pharmacists' professional profiles. This awareness not only strengthens students' identities but also their societal responsibility. The Portuguese Pharmaceutical Society emphasizes environmental responsibility, and incorporating this into education reflects institutional commitment and prepares professionals for regulatory demands. International guidelines emphasize the importance of sustainable practices in curricula, with the FIP recognizing pharmacists' role in mitigating ecological impact. The World Health Organization advocates for health professionals to lead the transition to sustainable health systems, emphasizing the importance of incorporating environmental awareness into education (50, 117).

## 5 Best practices for sustainable action

Rational use and proper disposal of medicines are essential for optimizing public health. Effective management ensures therapeutic efficacy and minimizes adverse impacts. Analyzing good sustainable pharmaceutical practices is crucial, as is highlighting the role of pharmacists and green pharmacy. Environmental education is vital for raising awareness and changing behaviors. Such initiatives require a robust regulatory framework, as reflected in sustainable international policies that guide an efficient pharmaceutical sector model. All these approaches should be included in the pharmaceutical curriculum.

### 5.1 Rational use of medicines

Rational use of medicines (RUM) is a key global health concept that promotes public health and efficient use of resources. According to the WHO, RUM occurs when patients receive appropriate medicines for their clinical needs, at the correct dosage, for the proper duration, and at an acceptable cost to both the patient and the community. Understanding this concept is crucial for preventing waste and adverse effects and promoting effective treatments. This definition emphasizes the importance of balancing clinical efficacy, safety, accessibility, and costs. In contrast, irrational use includes practices such as self-medication, excessive or inappropriate prescribing, the unnecessary use of antibiotics, and non-adherence to therapy. These practices compromise individual health and burden health systems with avoidable costs (134, 135).

Promoting RUM directly contributes to the sustainability of health systems by reducing the following: (i) the waste of financial resources on unnecessary medicines; (ii) the occurrence of preventable adverse events and subsequent hospitalisations; (iii) the progression of antimicrobial resistance, which requires longer, more expensive and less effective treatments (94).

Pharmacists play a key role in implementing RUM. As health professionals accessible to the community, they are involved in the revision of prescribed medication to identify interactions and duplications, advising on the correct use of drugs, treatment schedules, and duration, and the management of adverse effects; and raising awareness of the risks of self-medication and the importance of adhering to prescribed treatment. Studies show that educational pharmaceutical interventions can significantly increase adherence to therapy and reduce risky behaviors related to medication use (136).

For pharmacists to fulfill this role adequately, the academic curriculum must incorporate comprehensive content on rational pharmacotherapy, pharmacovigilance, patient communication, and health technology assessment. In addition to initial training, continuing education on new therapeutic guidelines, scientific updates, and health policies is crucial. This training enables pharmacists to practice critically and based on evidence, actively contributing to more sustainable therapeutic decisions (94, 117, 136).

Ultimately, equipping pharmacists with ongoing education and evidence-based practices not only improves patient outcomes but also cultivates a culture of sustainability in healthcare by encouraging the judicious use of medications and resources.

### 5.2 Proper disposal of medicines

Studies show that many people are unaware of how to dispose of unused medicines properly, making it essential for pharmacists to provide clear and practical information. Educational campaigns in pharmacies, along with explanatory materials on returns and collection points for pharmaceutical waste, are necessary to promote safe disposal methods. Furthermore, pharmacists should be involved in developing policies for the safe disposal of medicines and in participating in committees that develop guidelines and promote best community practices. Legislation on disposal should be incorporated into the health system, with pharmacists contributing their expertise. Collaborating with health authorities is crucial to establishing effective and viable environmental protection protocols, including collection programmes coordinated by pharmacists (137, 138).

Additionally, the literature review suggests that the primary method by which individuals from various nations dispose of unused or expired medications is either to throw them in regular household trash or flush them down the toilet. This pattern persists even in countries that have implemented programs to return surplus medications, suggesting that these initiatives are not sufficiently effective. Several factors may contribute to this issue, including a lack of education or ineffective awareness efforts within the community (139).

However, some countries have developed structured programs to address this problem by involving pharmacists directly in the medication disposal process. The Valormed programme is a prime example of how pharmacists are involved in waste management in Portugal. It focuses on ensuring that medicines are disposed of safely to prevent environmental contamination. Authorized pharmacies serve as return points for expired or unused medication, and public awareness campaigns also promote this service. Studies indicate that Valormed has increased the return rate, with pharmacists informing the public about the benefits of proper disposal methods (140).

### 5.3 Green pharmacy

Green Pharmacy is a movement that integrates sustainable practices into the pharmaceutical industry, aiming to reduce the environmental impact of pharmaceutical production. The focus is on re-evaluating the product life cycle, from development to disposal. The 12 principles of green chemistry inform the design of safer medicines. Reducing inputs begins with adopting “green chemistry” practices, which aim to minimize the use of hazardous substances and optimize chemical reactions. This includes using fewer toxic alternatives and biodegradable solvents, as well as continuous processes that improve yields and minimize by-products. Workspaces, such as laboratories and factories, should incorporate eco-efficiency principles and use technologies, such as microreactors, to minimize reagent volumes and waste. Assessing the life cycle of products helps identify opportunities to improve sustainable practices and supplier relationships. Energy efficiency is paramount, involving low-consumption technologies, renewable energy sources, and automatic systems to conserve energy. Effective inventory management is another measure that can minimize waste and contribute to the sustainability of the green pharmacy (141–143).

**Figure 2:**
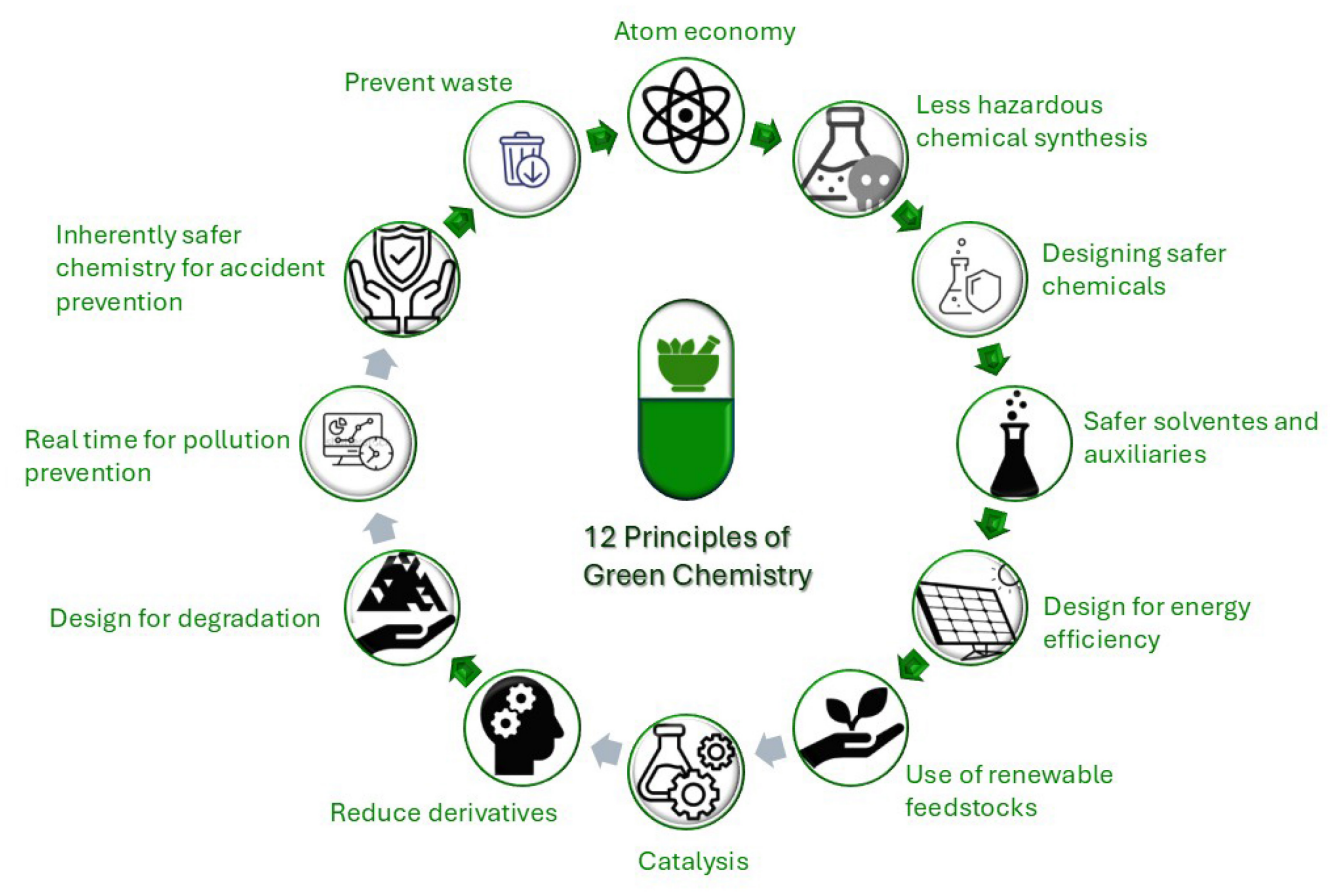
Twelve principles of green chemistry.

Figure 2 presents the 12 principles of green chemistry, which serve as a reference for designing safer and more sustainable medicines and processes throughout their life cycle.

The integration of green pharmacy concepts into pharmaceutical education curriculum enhances students' understanding of sustainable pharmaceutical practices and promotes the development of environmentally conscious healthcare professionals.

### 5.4 Environmental education for users and the community

Environmental education is a crucial component in shaping a society that understands and values environmental protection, particularly regarding the use and disposal of medicines. Pharmacies play a vital role in disseminating information that promotes responsible practices among users and the community. Integrating environmental education into daily pharmacy activities can positively influence population behavior and promote sustainability. Community pharmacies serve as a direct point of contact between health professionals and the community, making them a strategic platform for promoting health practices and environmental responsibility. Through environmental education, pharmacists can inform users about the environmental impacts of excessive consumption and improper disposal of medicines, as already mentioned.

In addition to training on the proper disposal of medicines, environmental education promotes conscious consumption practices, enabling pharmacists to advise users on the importance of not stockpiling unnecessary medication at home and of using only what is prescribed.

Environmental education programmes can be implemented through workshops, awareness campaigns, partnerships with community organizations, and lectures in schools. These are practical educational tools that enable the dissemination of information on environmental issues to a wide audience. When conducted in school settings, these presentations have the potential to influence students' attitudes and behaviors directly. Studies show that incorporating environmental education topics into school curricula is crucial for cultivating a generation that is more aware of ecological issues. Indonesia's Adiwiyata Programme, which aims to transform schools into environmentally responsible institutions, is a notable example that utilizes lectures as part of its awareness-raising strategy (144).

Through events, lectures, and informational materials, pharmacies can effectively influence community behavior. Several studies have shown that a lack of knowledge about safe practices is directly related to inappropriate behavior. Therefore, the creation of educational materials, such as leaflets and posters, distributed in pharmacies, can help transform users' misconceptions about various topics and contribute to the eradication of harmful environmental practices (130, 138, 145).

Pharmacists should be recognized as educators who can influence the community's attitudes and behaviors relating to health and the environment. Users currently place a high degree of trust in healthcare professionals, and interactions between the two groups are valuable opportunities for exchanging relevant information. By presenting themselves as reliable sources of information and providing practical guidance, pharmacists can encourage more sustainable behaviors (138).

It is therefore essential that pharmacists receive ongoing education on environmental and public health issues, enabling them to provide accurate and up-to-date information to their patients.

## 6 Challenges and perspectives for the future

### 6.1 Barriers to the implementation of sustainability in pharmaceutical education

**Figure 3:**
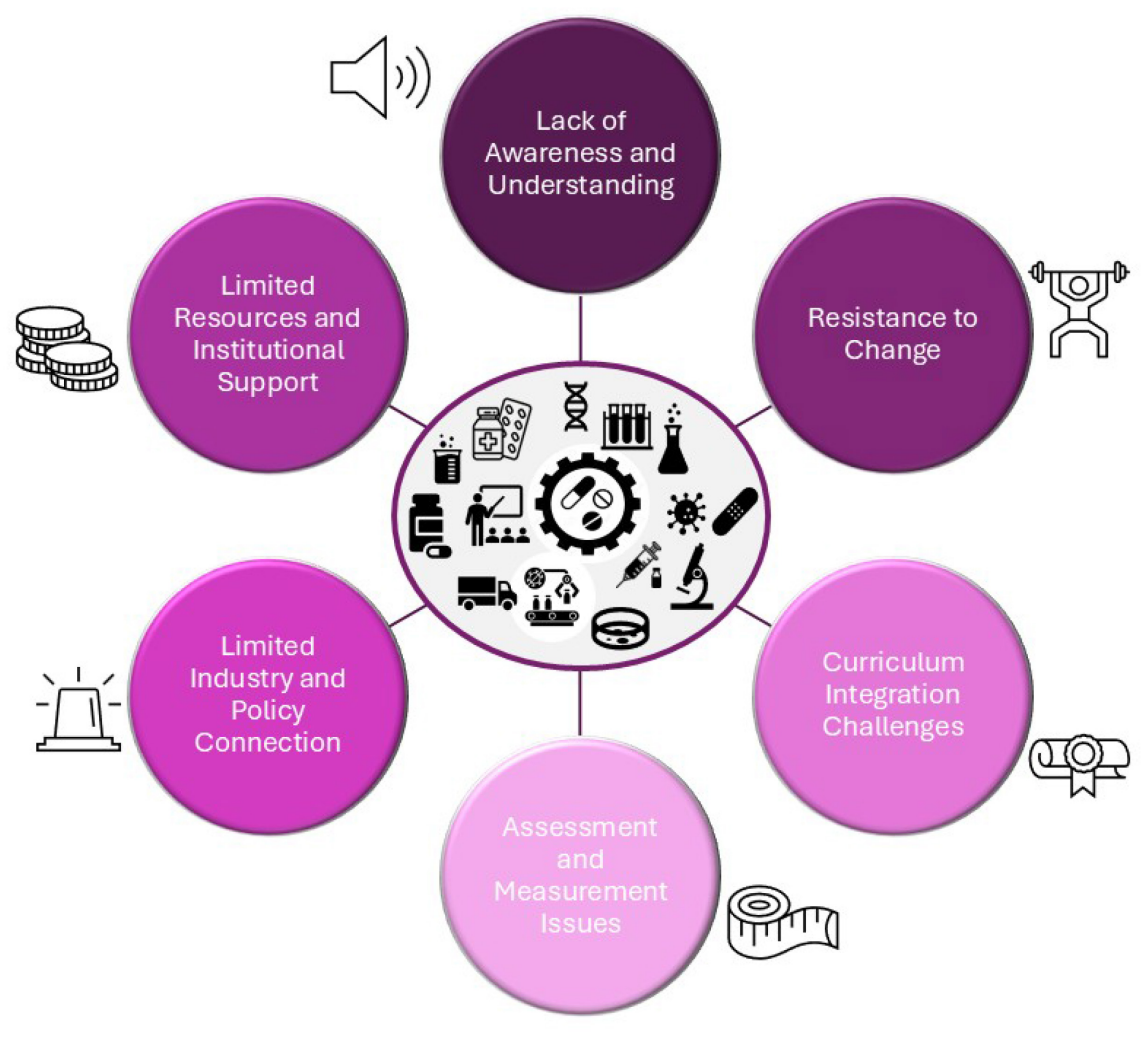
Barriers to learning sustainability in the pharmaceutical curriculum.

Although pharmaceutical education is vital for the sector's sustainability, the development of effective sustainability curricula is hindered by a lack of structured, integrated curricula that comprehensively address sustainability. Figure 3 shows the main barriers to addressing sustainability in pharmaceutical training.

The resistance from educational institutions to change existing curricula is often cited as a significant barrier. In many cases, the curriculum remains centered on traditional content, without including topics related to sustainability, such as environmental impacts, ethics, and social responsibility in pharmaceutical practice. Moreover, the absence of clear guidelines and implementation models in pharmaceutical education, especially in undergraduate courses, makes it difficult for institutions to implement significant changes (146).

One of the primary obstacles to promoting sustainability in pharmaceutical science education is the lack of awareness and understanding of the subject among both students and educators. Many learners have limited knowledge of what sustainability means in the context of pharmaceutical practice and often see it as a peripheral issue rather than an integral part of their professional responsibilities. Furthermore, sustainability is sometimes considered irrelevant to traditional pharmaceutical education, which has historically focused on subjects such as biology, chemistry, formulation and technology science, and clinical pharmacology (5).

A second major obstacle is the challenge of integrating the curriculum. In many institutions, sustainability-related topics are not embedded within core courses but are instead offered as optional or elective components. This limited inclusion reduces their perceived importance and impact. Furthermore, overcrowded curricula make it challenging to introduce new sustainability content without displacing foundational scientific subjects. Another issue is the lack of interdisciplinary collaboration between departments such as environmental science, pharmacology, and health policy. This hinders the development of comprehensive sustainability frameworks in pharmaceutical education (147). Moreover, the lack of interconnection between the curricular units also represents another barrier in pharmaceutical education. Frequently, courses are organized in ways that avoid interdisciplinary discussions of sustainability, creating compartmentalized knowledge. This phenomenon not only hinders a holistic understanding of the topics but also limits opportunities for collaboration across different areas of expertise, which are fundamental to promoting sustainability (148).

Another significant barrier is the limited availability of resources and institutional support. Many programmes struggle to secure adequate funding for sustainability-focused research, teaching materials, and student projects. Many institutions face budgetary constraints that limit their ability to develop new courses or provide professional development training for educators. Furthermore, there are few institutional incentives for faculty members to create or update teaching content related to sustainable practices. The lack of specialized facilities, such as sustainability-oriented laboratories or real-world case studies, further restricts students' practical learning opportunities (149).

Resistance to change within academic environments also hinders progress toward integrating sustainability principles. Teachers and institutions may be reluctant to modify established teaching methods or syllabuses, often perceiving sustainability as a “soft” or non-scientific topic compared to more traditional subjects, such as pharmacokinetics or medicinal chemistry. This cultural inertia in academia results in the slow adoption of innovative, sustainability-focused educational practices. Moreover, many teachers may not feel confident in teaching these new concepts, especially if they have not received adequate training on sustainability in their own classes (11, 150).

Students' perceptions of the importance of sustainability can also influence the effectiveness of the training. Many students may not fully understand the relevance of sustainability to their education and future professional practice, and this lack of awareness can lead to resistance to learning concepts that are not perceived as directly applicable to their future work as pharmacists (11, 148, 149).

External motivational factors, such as the need to meet curricular requirements or achieve good grades, can divert students' attention from meaningful, critical learning about sustainability. The hours devoted to teaching sustainable practices in courses are fewer than those dedicated to traditional areas of pharmacology, which may diminish students' perception of the necessity and priorities of these topics (146).

At a systemic level, connections between academia, industry, and policy are limited. Poor links with the pharmaceutical industry limit opportunities for collaboration on sustainable manufacturing, waste management, and supply chain practices. Furthermore, a lack of regulatory incentives and a scarcity of real-world role models demonstrating sustainable pharmaceutical systems reduces the visibility and perceived value of sustainability within the profession.

Finally, issues surrounding assessment and measurement pose additional challenges. There are no universally accepted metrics or indicators with which to evaluate sustainability learning outcomes within pharmaceutical education. Consequently, the impact of sustainability-focused interventions, such as curriculum changes or training programmes, remains difficult to quantify and assess effectively (151).

The lack of evaluation criteria and the exclusion of sustainability from the accreditation processes of pharmaceutical science courses are also significant barriers to consider. Currently, most accreditation standards focus on the technical and scientific aspects of the profession, neglecting the importance of sustainability training. This aspect highlights a missed opportunity, as modifying these criteria could encourage institutions to prioritize the inclusion of sustainability content in their curricula. Developing assessment practices that incorporate sustainability will enable institutions to encourage and reward teachers who introduce these topics in their classes. Moreover, a more comprehensive assessment can ensure that students graduate with a critical understanding of the challenges and sustainable solutions in pharmacology (11, 148, 149).

Many barriers to sustainable training in the pharmaceutical field arise from an institutional culture that undervalues sustainability initiatives, influenced by internal faculty mindsets and external market pressures and regulations. Promoting effective change requires fostering an educational culture that prioritizes sustainability (152).

### 6.2 Proposals to include more environmental content in pharmacy courses

Pharmaceutical education must evolve to integrate sustainable practices and environmental content, reflecting the growing importance of sustainability in public health and resource management.

**Figure 4:**
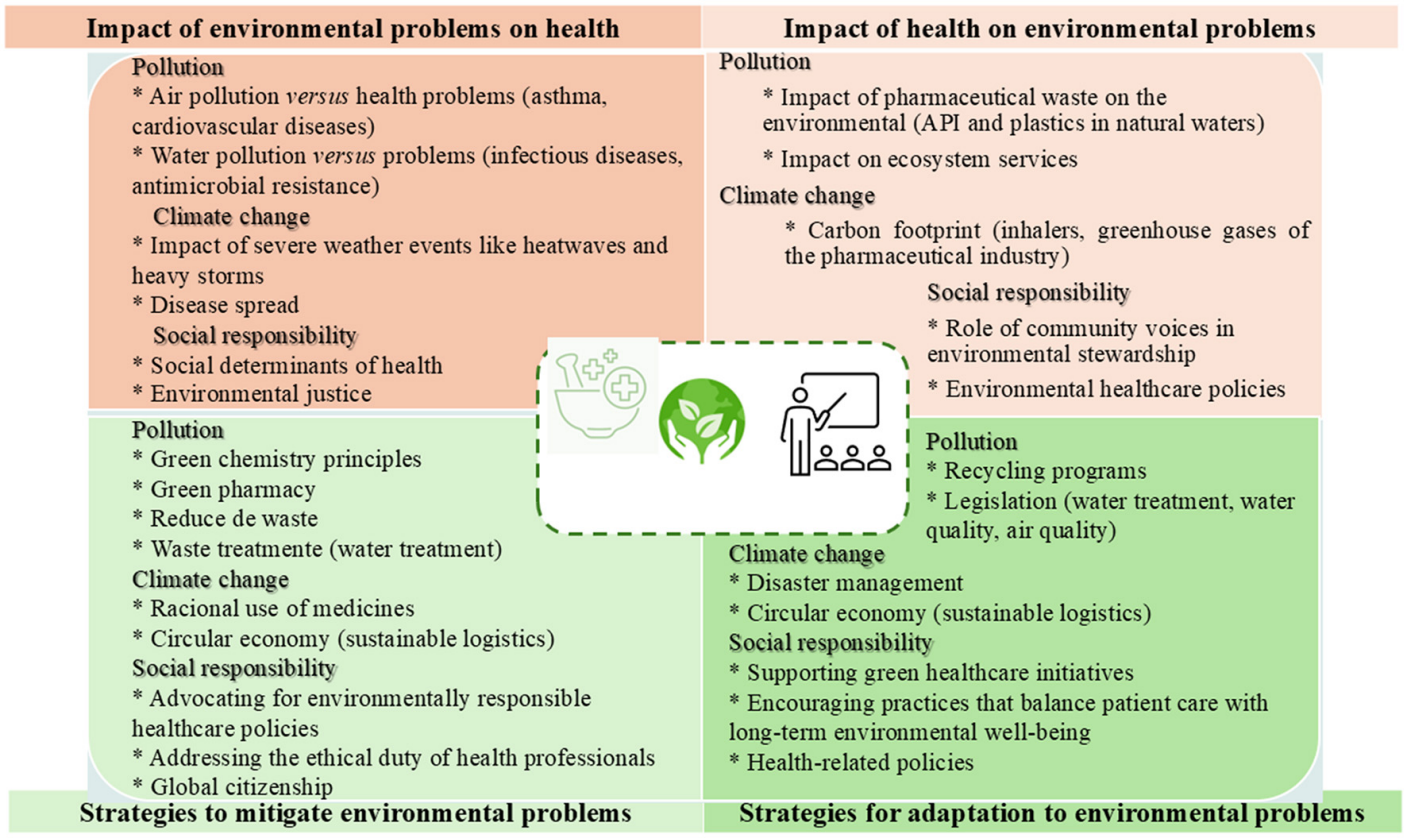
Proposed issues for pharmaceutical education relative to sustainability.

To strengthen environmental awareness in pharmacy education, courses should integrate content on pharmaceutical pollution, green chemistry, and sustainable prescribing practices. Interdisciplinary learning opportunities and experiential projects can help students connect planetary health principles with real-world healthcare challenges. Embedding sustainability competencies and indigenous perspectives into the curriculum will prepare future pharmacists to be leaders in environmental stewardship and social accountability (153). Figure 4 provides an overview of the sustainability-related themes to be included in pharmacist training, emphasizing the interconnection between environmental issues and human health.

The text highlights two reciprocal relationships: how environmental changes impact health, and vice versa. Environmental factors, including pollution and climate change, contribute to health issues such as respiratory problems and heat-related illnesses. On the other hand, healthcare practices contribute to environmental degradation. Strategies for addressing these issues include mitigation efforts, such as green chemistry and reducing pharmaceutical waste, as well as adaptation initiatives, including recycling and disaster management. Both approaches stress social responsibility, community involvement, and environmental justice, with education and public policies playing vital roles in promoting sustainable health and global citizenship.

The adoption of active teaching methodologies can also enhance learning about sustainability in Pharmacy. Courses should implement methods that encourage active student participation, such as case studies, community intervention projects, problem-based learning, and simulations of practical applications related to pharmaceutical sustainability (154).

For example, developing projects in partnership with community pharmacies for sustainable waste management can provide students with valuable practical experience, involving them in discussions about sustainability and the environment, and increasing their awareness and motivation for sustainable practices in the future (155).

Encouraging research and community practice is essential to connect students with the socio-environmental reality. Proposals such as supervised internships in community pharmacies and hospitals, with a centralized focus on sustainable practices, can provide practical, conscious learning. Through these experiences, students can observe and contribute to the implementation of pharmaceutical services that minimize environmental impacts, such as medication disposal programs and the promotion of sustainable therapeutic alternatives. Solidarity and social responsibility are integral parts that must permeate the academic training of future pharmacists (156).

Continuous teacher training is also a fundamental pillar. Professors of Pharmaceutical Sciences courses should be trained in sustainable practices and Environmental Education, enabling them to effectively impart this knowledge (113). Participation in workshops, seminars, and courses on best sustainability practices is essential to keep the faculty up to date. Moreover, initiatives that promote the exchange of experiences between institutions that have already implemented successful sustainability programs can inspire others to do the same (157).

Promoting sustainability research should also be a central objective of Pharmacy schools. Incentives for research addressing environmental issues and their interactions with pharmacology could encourage students and faculty to pursue innovative, sustainable solutions. For example, developing new methods for manufacturing medications that minimize environmental impact can be a fundamental area of research. Furthermore, creating specific calls for proposals and funding lines for sustainability projects in the pharmaceutical industry is an alternative that would promote the inclusion of the topic in curricula and academic research (7).

The integration of environmental content into Pharmaceutical Sciences courses is essential for training professionals who are aware of and prepared to address contemporary environmental challenges. Through curriculum review, the implementation of active methodologies, encouragement of research, and teacher training, it is possible to create a pharmaceutical education that prioritizes sustainability and care for the health of the planet. Thus, the future professional will not only be a specialist in medications but also an active advocate for sustainable practices in the health sector.

## 7 Conclusions

The growing awareness around the global environmental crisis demands a profound reflection on the role of different professional areas in promoting sustainability.

The pharmaceutical industry has a significant ecological footprint, resulting from highly energy-intensive manufacturing processes, the emission of persistent chemical and pharmaceutical compounds, and the production of complex, difficult-to-treat waste. Despite advances in energy efficiency and technological innovation, gaps still exist in environmental regulation and the application of green chemistry principles.

At the same time, the training of future pharmaceutical professionals has proven to be a crucial axis for a paradigm shift. The inclusion of environmental sustainability content in the curricula of Pharmaceutical Sciences in Portugal and other countries remains incipient, underscoring the need to strengthen environmental literacy, ecological ethics, and students' practical training to equip them to act as agents of transformation in their professional contexts.

The good practices, such as rational medication use, green pharmacy, promoting collection systems, and eco-design of packaging, demonstrate pharmacists' potential to act in accordance with the Sustainable Development Goals. However, its full implementation requires a joint effort between universities, regulatory authorities, industry, pharmacies, laboratories, and civil society.

It is concluded, therefore, that promoting environmentally sustainable pharmaceutical practices is not just an ethical option but an urgent requirement in the face of the challenges of the 21st century. To this end, it is essential to integrate sustainability as a transversal and structuring dimension of the pharmaceutical profession, from academic training to professional practice, to ensure a healthcare system that respects the planet's ecological limits and promotes the wellbeing of present and future generations.

Universities are responsible for equipping students and future healthcare professionals to navigate the complexities of the healthcare system. However, pharmaceutical programs have typically overlooked the environmental considerations of pharmaceuticals and the systems-thinking approach to healthcare and pharmaceutical sciences. This revision paper presents an opportunity to consider the role of universities in addressing sustainable issues within their curricular units, encompassing all pharmaceutical activities.
